# Nanosecond Electric Pulse Effects on Gene Expression

**DOI:** 10.1007/s00232-013-9579-y

**Published:** 2013-07-06

**Authors:** Louise Chopinet, Tina Batista-Napotnik, Audrey Montigny, Matej Rebersek, Justin Teissié, Marie-Pierre Rols, Damijan Miklavčič

**Affiliations:** 1 CNRS, IPBS - UMR 5089, 205 route de Narbonne, BP 64182, 31077 Toulouse Cedex 4, France; 2 UT1, UTM, LAAS, ITAV, UPS, INSA, INP, ISAE, Université de Toulouse, 31077 Toulouse Cedex 4, France; 3 Faculty of Electrical Engineering, University of Ljubljana, Ljubljana, Slovenia

**Keywords:** Nanosecond electric pulse, Gene electrotransfection, Electroporation, Nuclear envelope, Plasmid DNA

## Abstract

Gene electrotransfection using micro- or millisecond electric pulses is a well-established method for safe gene transfer. For efficient transfection, plasmid DNA has to reach the nucleus. Shorter, high-intensity nanosecond electric pulses (nsEPs) affect internal cell membranes and may contribute to an increased uptake of plasmid by the nucleus. In our study, nsEPs were applied to Chinese hamster ovary (CHO) cells after classical gene electrotransfer, using micro- or millisecond pulses with a plasmid coding the green fluorescent protein (GFP). Time gaps between classical gene electrotransfer and nsEPs were varied (0.5, 2, 6 and 24 h) and three different nsEP parameters were used: 18 ns-10 kV/cm, 10 ns-40 kV/cm and 15 ns-60 kV/cm. Results analyzed by either fluorescence microscopy or flow cytometry showed that neither the percentage of electrotransfected cells nor the amount of GFP expressed was increased by nsEP. All nsEP parameters also had no effects on GFP fluorescence intensity of human colorectal tumor cells (HCT-116) with constitutive expression of GFP. We thus conclude that nsEPs have no major contribution to gene electrotransfer in CHO cells and no effect on constitutive GFP expression in HCT-116 cells.

## Introduction

Electroporation is a physical method used to improve delivery of nonpermeant molecules into cells. The technique was introduced by Neumann and Rosenheck (1972), and its mechanism has been studied for decades. It is used in clinics to potentiate the effects of cytotoxic drugs for cancer treatment, a method called “electrochemotherapy” (ECT) (Mir et al. 2003). Based on the use of “medium”-lasting electric pulses (100–900 μs at an electric field in the range of several hundreds of volts per centimeter), ECT permeabilizes the plasma membrane of tumor cells and allows anticancer drugs (such as bleomycin and cisplatin) to enter directly into the cytoplasm and eventually kill tumor cells (Kotnik et al. 2012). These medium pulses or longer ones (1–10 ms with an electric field intensity in the range of several hundred volts per centimeter) are also used for gene transfer as they not only permeabilize the membrane but also cause DNA to move toward the permabilized cell membrane due to electric forces (electrophoresis) and to enter the cell to be expressed (Escoffre et al. 2010; Golzio et al. 2002a; Kanduser et al. 2009; Paganin-Gioanni et al. 2011; Satkauskas et al. 2002). This approach, called “gene electrotransfer,” has a main clinical application in gene therapy and DNA vaccination, and for now one clinical trial has been published (Daud et al. 2008), while several others are ongoing (Heller and Heller 2010; El-Kamary et al. 2012). Longer (millisecond) pulses have been shown to be the more efficient in gene transfer (Cemazar et al. 2009). The protocol using classical gene electrotransfection parameters (8 × 5 ms, 700 V/cm, 1 Hz) is efficient in vitro since >30 % of cells can express the gene coded by plasmid DNA (Chinese hamster ovary [CHO] and human colorectal tumor [HCT] cells) while preserving cell viability to a large extent (Chopinet et al. 2012; Golzio et al. 2002a; this study). However, in skin tumors, this rate decreases dramatically both ex vivo (Chopinet et al. 2012) and in vivo (Cemazar et al. 2009; Rols et al. 1998). Improvements of the method are therefore needed to allow its wider use in gene therapy.

Gene transfer can be described as a two-barrier process at the cell level irrespective of vectorization technique (viral, chemical or physical). Firstly, plasmid DNA must cross the plasma membrane; and secondly, after migration through the cytoplasm, it must cross the nuclear envelope in order to be expressed. Studies on the cell cycle have shown that gene electrotransfer efficiency is increased when the nuclear envelope is disrupted, i.e., when cells are pulsed during G_2_ phase (Golzio et al. 2002b; Escoffre et al. 2010). Exposure of cells to medium and long electric pulses leads to plasma membrane permeabilization; thus, the first barrier is overcome, but the second barrier, the nuclear envelope, remains a challenge.

**Fig. 1:**
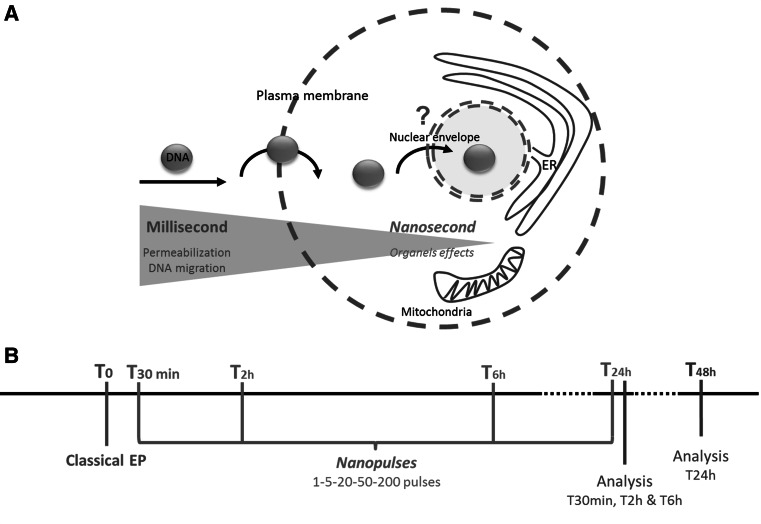
Biological context and experimental outline (Color figure online)

Deng et al. (2003) and Schoenbach et al. (2001) introduced a new class of short pulses into electroporation research, called “nanosecond electric pulses” (nsEPs, 4–600 ns), linked to technological improvements (Rebersek and Miklavcic 2011; Sundararajan 2009). These nsEPs are described as being able to disturb membranes of internal organelles under high voltage (several tens of kilovolts per centimeter). Numerical simulation as well as theory showed that nsEPs are capable of destabilizing internal cell membranes because of their charging time (Gowrishankar et al. 2011; Kotnik and Miklavcic 2006; Schoenbach et al. 2001; Tekle et al. 2005; Retelj et al. 2013). Results obtained in vitro revealed several effects on cell organelles such as permeabilization of intracellular granules (Schoenbach et al. 2001), endocytotic vesicles (Napotnik et al. 2010) and large endocytosed vacuoles (Tekle et al. 2005) as well as calcium release from endoplasmic reticulum (ER) (Beebe et al. 2003; for review, see Joshi and Schoenbach 2010). In this context, as nsEPs have an effect on internal organelle membranes, the point was to consider that they might have an effect on the nuclear envelope. Using nsEPs, the nuclear envelope barrier could be overcame and gene electrotransfer efficiency enhanced by allowing plasmid DNA (already present in the cell cytoplasm due to large pulses) to gain access to the nucleus. This is why over 10 years ago the following strategy began to be investigated: combination of medium or long electrical pulses to first permeabilize the plasma membrane and allow plasmid to access cytoplasm, followed by application of nsEPs to destabilize the nuclear envelope and enhance gene expression by increasing the number of plasmids entering the nucleus (Fig. 1a). Beebe et al. (2003) described that nsEPs may have a significant effect on gene electrotransfer. In this publication, a 3.6-fold increase in gene expression (green fluorescent protein [GFP] fluorescence intensity) was measured by flow cytometry for cells exposed to classical electroporation plus one nsEP 30 min later when compared to control with only classical electroporation. The same results were reported by Beebe et al. (2004).

Since then, however, no other publication can be found on this subject using nsEPs. One work using one pulse of 5-μs at 9 kV/cm showed some effects on the nucleus and permeability but not on gene transfection (Bellard and Teissié 2009). In our present study, we followed the same strategy with a new set of nsEPs using multiple pulses and different delays between classical EPs and nsEPs (Fig. 1b). We performed three sets of experiments with different electrical parameters but using the same protocol, meaning we first pulsed CHO cells in the presence of a plasmid coding the GFP with classical gene electrotransfer parameters and after different time gaps (0 and 30 min and 2, 6 and 24 h) applied 1, 5, 20, 50 or 200 nsEPs.

The first set (see Table 1) was composed of the combination of 8 × 5 ms, 400 V/cm, 1-Hz pulses for classical gene transfer and 18 ns, 10 kV/cm, 10 Hz for nsEPs. For the second set we used the same classical electrotransfection protocol and 10 ns, 40 kV/cm, 10 Hz for nsEPs. For the third and last set classical electrotransfection was performed with 4 × 200 μs, 1.2 kV/cm, 1 Hz and 15 ns, 60 kV/cm, 10 Hz was used for nsEPs. Then, 24 h after nsEP application we analyzed transfection rate and fluorescence intensity by flow cytometry or fluorescence microscopy (depending on the lab where the experiments were performed). We also exposed human colorectal tumor cells (HCT-116), stably transfected for GFP, to all nsEP parameters and analyzed the effect these might have on gene expression. Overall, no major effects of nsEPs could be detected.

**Table 1:** Description of the three sets of electrical parameters used

	Classical electrotransfection parameters	nsEP parameters
Set 1	8 × 5 ms, 400 V/cm, 1 Hz	18 ns, 10 kV/cm, 10 Hz
Set 2	8 × 5 ms, 400 V/cm, 1 Hz	10 ns, 40 kV/cm, 10 Hz
Set 3	4 × 200 μs, 1.2 kV/cm, 1 Hz	15 ns, 60 kV/cm, 10 Hz

## Materials and Methods

### Cell Culture

CHO cells (wild-type Toronto; ATCC, Manassas, VA) were used for studying gene electrotransfection and nsEPs. Cells were grown as a monolayer culture in minimum essential Eagle medium with Earle’s salts and nonessential amino acids (EMEM; Eurobio, Les Ulis, France), supplemented with 10 % fetal bovine serum (GIBCO/Life Technologies, Grand Island, NY), l-glutamine (0.58 g/l, GIBCO/Life Technologies), 2.95 g/l tryptose-phosphate (Sigma-Aldrich, St. Louis, MO), BME vitamins (Sigma-Aldrich), 3.5 g/l glucose (Sigma-Aldrich) and the antibiotics penicillin (100 U/ml) and streptomycin (100 μg/ml, both from GIBCO/Life Technologies) at 37 °C, 5 % CO_2_ atmosphere in a humidified chamber until they reached 70 % confluence.

HCT-116 cells, which are derived from human colorectal carcinoma cells and present constitutive expression of GFP, were used for studying the effects of nsEPs on gene expression. Cells were infected with viral vectors to stably express enhanced GFP (eGFP). To that purpose, a retroviral vector, MFG-eGFP, encoding eGFP under the control of 50 long terminal repeats (LTRs), was used. 293T cells, generously provided by Genethon (Evry, France), were transiently transducted using the calcium phosphate coprecipitation protocol with pMDG encoding VSV-G protein, pGagPol encoding gag and pol and MFG-eGFP. Viruses containing supernatants were collected 36–72 h after transduction, filtered and concentrated to titers of 1 to 5 × 10^9^ colony forming units/ml. HCT-116 cells were plated in a 35-mm culture dish 24 h prior to transduction. On day 0 cells were transduced with viral vectors at a multiplicity of infection of 100:1. After transduction (48 h), cells were harvested for FACS analysis on a Becton–Dickinson FacsCalibur to select cells stably expressing eGFP. Cells were grown as a monolayer culture in Dulbecco’s modified Eagle medium with glucose, l-glutamine and sodium pyruvate (GIBCO/Life Technologies), supplemented with 10 % fetal bovine serum and the antibiotics penicillin and streptomycin at 37 °C, 5 % CO_2_ atmosphere in a humidified chamber.

### Gene Electrotransfection Protocols

Three different conditions were used to study nsEP effects on gene transfer, using two different electrical parameters for classical gene electrotransfer and three different electrical parameters for nsEPs as summarized in Table 1. Each experiment was repeated three times independently.

CHO cells were first exposed to electric pulses that are generally used in gene transfer electroporation (EP) protocols. Cells were incubated for 0 and 30 min and for 2, 6 and 24 h and then exposed to nsEPs (1, 5, 20, 50 and 200 pulses). Cells were trypsinized and suspended in phosphate buffer (PB; 10 mM KH_2_PO_4_/KH_2_PO_4_, 1 mM MgCl_2_, 250 mM saccharose [pH 7.4]) at a concentration of 3 × 10^6^ cells/ml, and 40 μg/ml pEGFP-C1 plasmid was added. For 8 × 5 ms, 400 V, 1-Hz parameters 420 μl of cell solution was put between stainless steel, flat, parallel electrodes (1-cm gap, resulting in an electric field of 400 V/cm) and exposed to square-wave electric pulses at room temperature using a pulse generator (electrocellS20; Betatech, Bordeaux, France). For 4 × 200 μs, 1 Hz, 480 V parameters, 800 μl of cell suspension was placed into an Eppendorf (Hamburg, Germany) cuvette (4 mm, resulting in an electric field of 1.2 kV/cm), and pEGFP-N1 plasmid was added (40 μg/ml). Cells were electroporated with an electric pulse generator (GHT 1287B; Jouan, St. Herblain, France).

For 0 min incubation, cells were immediately transferred to electroporation cuvettes and pulsed with nsEPs (see “Nanosecond Electroporation” below). For longer incubation times, fetal bovine serum was added to cells after pulsing (20 % of suspension volume), and the mixture was incubated for 5 min at 37 °C to prevent cells from dying and to improve plasma membrane resealing (Delteil et al. 2000; Haberl et al. 2010). Cells were then transferred to 5 ml EMEM and incubated for 30 min or 2 h, with occasional shaking. Cells that were incubated for 6 and 24 h were seeded to a small culture flask (25 cm^2^) and placed in a 5 % CO_2_ incubator, allowing them to attach to the surface; later they were trypsinized, centrifuged and resuspended in 70 μl of pulsation buffer for nsEP.

### Nanosecond Electroporation

Cells in EMEM were centrifuged and transferred to PB at a concentration of 3 × 10^6^ cells/ml. Cell suspension (70 μl) was placed in electroporation cuvettes with built-in aluminum electrodes with a 1-mm gap (Eppendorf). Cells were pulsed with 1, 5, 20, 50 and 200 nsEPs. Cells pulsed only with classical EP pulses, no nsEPs, were used as a control. After applying nsEPs, fetal bovine serum was added to cells after pulsing (20 % of suspension volume) and incubated for 5 min. Finally, cells were placed in 24-well plates in 1 ml of medium and incubated for 24 h (from EP pulsing) in a CO_2_ chamber.

At 24 h after nsEP pulsing, cells were analyzed by flow cytometry or fluorescence microscopy (see below). For the point where nsEPs are applied 24 h after classical parameters, GFP expression was analyzed 24 h after nsEP application.

### Plasmid DNA

pEGFP-C1 or pEGFP-N1 (Clontech, Mountain View, CA), a 4.7-kb plasmid DNA encoding GFP, was amplified in *Escherichia coli* DH5α and purified with the Maxiprep DNA Purification System or the HiSpeed Maxi kit (Qiagen, Hilden, Germany) according to the manufacturer’s protocol.

### nsEP Generators

**Fig. 2:**
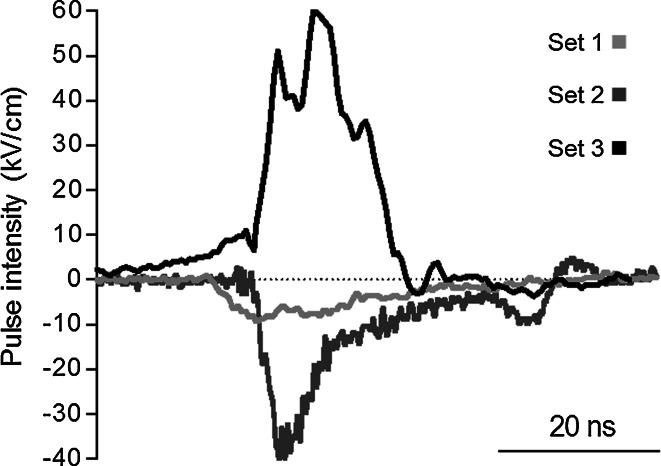
nsEP profiles for the three sets described in Table 1

A PBG2 (Kentech Instruments Ltd, Wallingford, UK) generator was used, delivering 18- and 10-ns pulses at 10 or 40 kV/cm, respectively (sets 1 and 2) (Fig. 2). The pulse profile was recorded directly on the electrodes through a Barth attenuator (142-HMFP-10 dB; Barth, Boulder City, NV) and other attenuatuors that on the whole attenuate up to 60 dB (Kenaan et al. 2011) by means of an oscilloscope (TDS5104B, 1 GHz; Tektronix, Beaverton, OR). This generator was triggered by a Betatech generator (Electrocell S20) to monitor the number of pulses and frequency.

For the third set of nsEP parameters, a custom-designed nanosecond, high-voltage electric pulse generator was used. It was designed and manufactured at the Laboratory of Biocybernetics at the Faculty of Electrical Engineering, University of Ljubljana, as a diode opening switch generator, described elsewhere (Rebersek and Miklavcic 2011). The pulses were measured at the electrodes by a LeCroy PPE 6 kV probe and the Wave Surfer 422 oscilloscope (Teledyne LeCroy, Chestnut Ridge, NY) (Fig. 2).

### Permeabilization Assay

Cells were pulsed in the conditions described above (pulsing protocol one) in the presence of 0.1 mM propidium iodide (Sigma-Aldrich), incubated for 5 min at room temperature and observed under a fluorescence microscope (Leica, Wetzlar, Germany; DMRIB microscope, filter 515–560, Mirror 580, LP 590, 100× objective for permeabilization observation).

### Gene Expression

HCT-116 cells with constitutive expression of GFP were trypsinized and suspended in PB at a concentration of 3 × 10^6^ cells/ml. Cells were then transferred to electroporation cuvettes, and 1, 5, 20, 50 and 200 nsEPs were applied with the three set of parameters described previously. Cells were then incubated for 24 h in the same manner as CHO cells.

### Flow Cytometry and Data Analysis (Sets 1 and 2)

At 24 h after EP application (for sets 1 and 2) cells were trypsinized and suspended in Dulbecco’s phosphate-buffered saline without Mg and Ca (Eurobio). Cells with a 24-h time gap between EP and nsEP were trypsinized 24 h after nsEP (48 h after EP). Cells were then analyzed by flow cytometry at λEX = 488 nm and λEM = 520/42 nm BP (FacsCalibur, Becton–Dickinson). A minimum of 2,000 cells (debris excluded) were counted per sample. Data were analyzed using Excel software (Microsoft, Redmond, WA). The mean fluorescence of transfected cells was normalized to the control for figure presentation. One-way ANOVA repeated measurement was used on raw data to determine statistical differences between pulsed groups and control using GraphPad Prism Software (GraphPad Software, La Jolla, CA).

### Fluorescence Microscopy (Set 3)

After 24 h, three images per Petri dish on a distinct area were recorded using an epifluorescent microscope (Leica DFC450 C): 40× objective, excitation wavelength 470 nm and appropriate filter set (EX470/D495/EM525), with the same image acquisition parameters (for transfection, CHO cells, exposure time 1.5 s, gain 5×; for expression, HCT-116 cells, exposition time 1 s, gain, 4×). Images were analyzed with a Java-based image processing program (ImageJ; National Institutes of Health, Bethesda, MD); fluorescent cells were counted and the mean fluorescence of cells was determined (background fluorescence subtracted). Statistical analysis was performed using Excel and SygmaPlot (Systat Software, Chicago, IL). Statistically significant differences were tested using one-way ANOVA.

## Results and Discussion

### Do nsEPs Improve Plasmid DNA Nuclear Envelope Crossing?

**Fig. 3:**
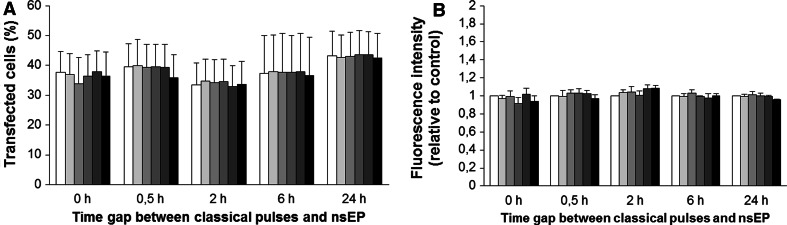
Effect of nsEP on gene electrotransfection in CHO cells—set 1 parameters. *White bar* represents control and progressively *grey bars* represent 1, 5, 20 and 50 nsEP, with the *black bar* representing 200 nsEP. **a** Percentage of transfected cells. **b** Mean GFP fluorescence intensity relative to control in transfected cells. *Data* are means ± standard errors of three independent experiments

**Fig. 4:**
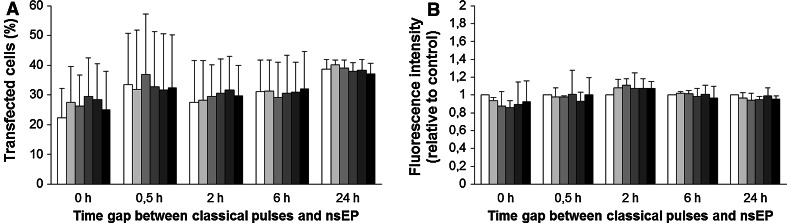
Effect of nsEP on gene electrotransfection in CHO cells—set 2 parameters. *White bar* represents control and progressively *grey bars* represent 1, 5, 20 and 50 nsEP, with the *black bar* representing 200 nsEP. **a** Percentage of transfected cells. **b** Mean GFP fluorescence intensity relative to control in transfected cells. *Data* are means ± standard errors of three independent experiments

**Fig. 5:**
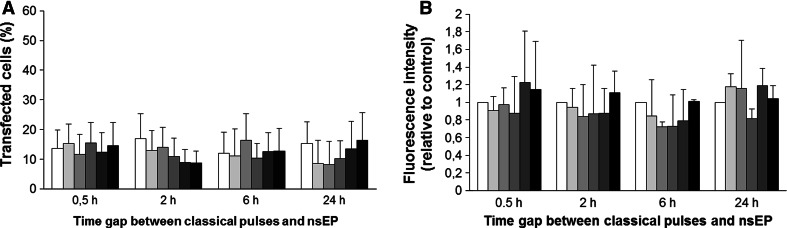
Effect of nsEP on gene electrotransfection in CHO cells—set 3 parameters. *White bar* represents control and progressively *grey bars* represent 1, 5, 20 and 50 nsEP, with the *black bar* representing 200 nsEP. **a** Percentage of transfected cells. **b** Mean GFP fluorescence intensity relative to control in transfected cells. *Data* are means ± standard errors of three independent experiments

CHO cells were pulsed in the presence of plasmid DNA with a combination of classical EP pulses and nsEP. Time gaps between classical EP and nsEP pulses (0 and 30 min, 2, 6 and 24 h) and number of nsEPs (0, 1, 5, 20, 50 and 200) were varied. We used three different sets of parameters (Table 1). At 24 h after nsEP application gene expression was determined either by flow cytometry (sets 1 and 2, Figs. 3, 4) or by fluorescence microscopy (set 3, Fig. 5). Compared to data obtained only for classical electrotransfection pulse application (0 nsEP), results show that transfection rates (% of transfected cells) in control cells (submitted only to classical EP) present a mean of 31 ± 11 % using sets 1 and 2 (Figs. 3a, 4a) and 14 ± 8 % in set 3 (Fig. 5a). These results demonstrate that, depending on the plasmid used and the classical electrotransfer parameters chosen, the transfection rate is different, also depending on the use of millisecond pulses (sets 1 and 2) or microsecond pulses (set 3) in vitro, though both well within the range usually reported for these kinds of electric parameters.

**Fig. 6:**
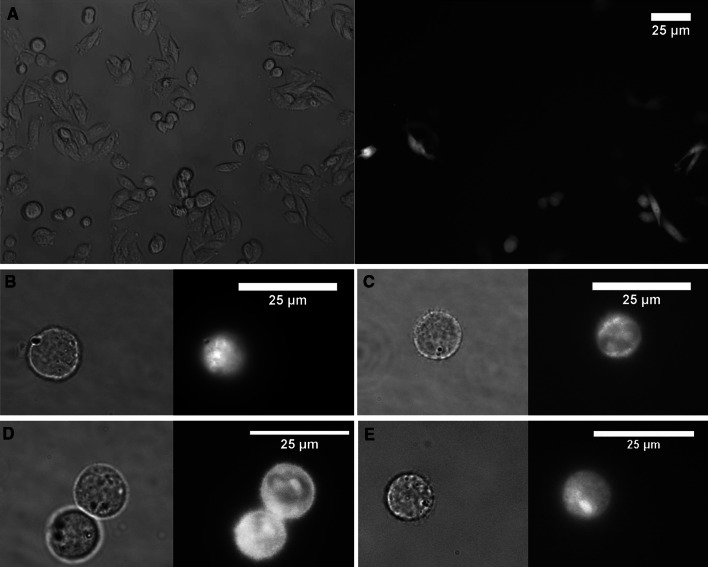
Expression and permeabilization of cells. **a** CHO cells to set 1 parameters in 6-h time gap between EP and nsEP, observed 24 h after nsEP. Phase contrast image and fluorescence image of GFP expression (40× objective). **b** Permeabilization of cells observed by entry of propidium iodide, contrast phase and fluorescence image for classical electroporation protocol only of set 1 (100× objective). **c**–**e** Permeabilization of cells submitted to 200 nsEP only of sets 1, 2 and 3, respectively—phase contrast image and fluorescence-associated (100× objective)

No significant effects on the percentage of GFP-positive cells can be measured when cells were submitted to nsEPs immediately after the classical EP (Figs. 3a, 4a, time 0). Despite the fact that nsEPs with these electrical parameters permeabilize the plasma membrane (Fig. 6c–e), these results are consistent with the fact that plasmids can only enter the cell when classical EP is applied as no increase in transfection rate is observed when nsEPs are applied immediately after classical EP. Indeed, DNA needs electrophoretic forces to migrate toward the permeabilized plasma membrane and enter the cell (Faurie et al. 2010; Kanduser et al. 2009). This means that nsEPs do not participate in plasma membrane permeabilization in the same way that classical EP does and do not allow DNA to cross the plasma membrane. Thus, for the last set (i.e., set 3) of electrical conditions, we performed nsEP only at 30 min and 2, 6 and 24 h after classical EP.

When the nsEPs were applied at different time gaps after classical EP (0, 5, 2, 6 and 24 h), no increase in transfection rate was obtained (Figs. 3a, 4a, 5a). As no DNA was added for these points, we were expecting an increase in the number of plasmids expressed (fluorescence intensity) more than the transfection rate. When analyzing the mean fluorescence intensity of transfected cells, no statistical increase in gene expression was measured whatever the nsEP parameters (Figs. 3b, 4b, 5b).

Fluorescence intensity is directly correlated to the number of GFPs inside the cell, linked to the number of expressed gene, which means the number of plasmids inside the cell that have reached the nucleus transcriptional machinery (Cohen et al. 2009). As we know, DNA must go from the plasma membrane to the nucleus through the cytoplasm. This involves active transportation of plasmid DNA (Rosazza et al. 2011, 2012; Vaughan and Dean 2006). Here, we show that no further increase in the amount of DNA accessing the nucleus, and therefore protein expression, can be detected when 200 nsEPs are applied 2 or 6 h after classical EP (i.e., after plasmid DNA is already present inside the cell). Thus, at these times, even if there is still DNA in the cytoplasm but not yet in the nucleus, nsEPs do not have a beneficial effect on nuclear envelope crossing. The effect of endonuclease activity and further inactivation of plasmid can also be taken into account for this absence of effect. Moreover, as shown in 2009 and 2011, nsEPs are able to trigger an actin response in plant cells, so these effects on cytoskeletal components can interfere with DNA migration and thus stop its motion toward to the nucleus (Berghöfer et al. 2009; Hohenberger et al. 2011).

In contrast to the results reported by Beebe et al. (2003, 2004), we observed no increase after applying a single nsEP or up to 200 nsEPs, independently of the time after classical EP that they were applied as well as electrical parameters. Even if the intensity in our study is lower than that in the studies by Beebe et al., we expected that applying a large number of pulses should have had an effect. In addition, nsEPs did not contribute to a better transfection rate—the percentage of transfected cells remained unchanged. Numerical simulations (Joshi et al. 2004; Kotnik and Miklavcic 2006; Retelj et al. 2013) predict that the electric parameters of cells and organelles (e.g., membrane capacitance and internal conductivity) lead to differences in charging times of the membranes, organelles presenting a shorter time than the plasma membrane and thus are potentially specifically sensible to nsEP. The plasma membrane is a simple lipid bilayer, whereas the nuclear envelope consists of two lipid bilayers. The outer bilayer is connected to the ER and the inner is supported by nuclear lamina and DNA, which confer to the nuclear envelope a higher complexity than ER or mitochondria that has been shown to be destabilized by nsEP. Moreover, it has several proteins inserted in and large pore complexes that span through both bilayers (Wente and Rout 2010) that may prevent any effects of electric fields as the nuclear envelope cannot represent a simple capacitor. Nowadays, no simulations at this level of complexity are available in the literature, and therefore, only experimental data can help us to define nsEP incidence on this structure.

### Do nsEPs Affect Endogenous Expression?

**Fig. 7:**
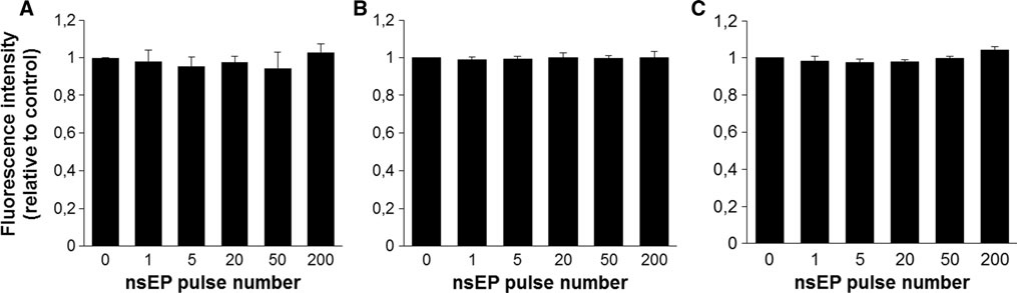
Effect of nsEP on expression of GFP in HCT-116-GFP cells: mean GFP fluorescence intensity relative to control is presented. *Data* are means ± standard errors of three independent experiments. **a** Results 24 h after nsEP of 18 ns, 10 kV/cm, 10 Hz. **b** Results 24 h after nsEP of 10 ns, 40 kV/cm, 10 Hz. **c** Results 24 h after nsEP of 15 ns, 60 kV/cm, 10 Hz

HCT-116 cells with constitutive expression of GFP were pulsed using the three electrical parameters for nsEP as presented in Table 1 but without any classical pulses applied prior to nsEP. Mean GFP fluorescence was compared to nonpulsed control cells. Applying nsEP did not alter mean fluorescence compared to nonpulsed cells as measured by flow cytometry (Fig. 7a, b) or fluorescence microscopy (Fig. 7c). In all of these conditions, no effect could be measured. These results suggest that nsEP has no effect on the cell lines used in our experiments for GFP expression (CHO transient electrotransfection and HCT endogenous expression).

## Conclusion

We can conclude that, under all conditions used in our study, nsEPs have no effect on gene expression (either for transfected or for endogenous genes) and their use, according to the present knowledge and experience, will not help in increasing gene electrotransfection efficiency. We can state that nsEPs are not “permeabilizing,” i.e., breaching the nuclear envelope or plasma membrane in the same way as classical EP does with long and medium pulses that efficiently permeabilize the plasma membrane. Other experiments performed with a lower quantity of plasmid or with a higher electric field intensity and number of pulses could perhaps help to detect an effect of nsEP on the nuclear envelope by making an increase in the number of plasmids expressed more visible.
